# Chemical vapor-deposited carbon nanofibers on carbon fabric for supercapacitor electrode applications

**DOI:** 10.1186/1556-276X-7-651

**Published:** 2012-11-27

**Authors:** Yang Gao, Gaind P Pandey, James Turner, Charles R Westgate, Bahgat Sammakia

**Affiliations:** 1 Mechanical Engineering Department, Binghamton University, State University of New York, Binghamton, NY, 13902, USA; 2 Center for Autonomous Solar Power (CASP), Binghamton University, State University of New York, Binghamton, NY, 13902, USA; 3 Small Scale Systems Integration and Packaging Center (S3IP), Binghamton University, State University of New York, Binghamton, NY, 13902, USA

**Keywords:** Carbon nanofibers, Carbon fabric, Water-assisted chemical vapor deposition, Supercapacitor, Cyclic voltammetry

## Abstract

Entangled carbon nanofibers (CNFs) were synthesized on a flexible carbon fabric (CF) via water-assisted chemical vapor deposition at 800°C at atmospheric pressure utilizing iron (Fe) nanoparticles as catalysts, ethylene (C_2_H_4_) as the precursor gas, and argon (Ar) and hydrogen (H_2_) as the carrier gases. Scanning electron microscopy, transmission electron microscopy, and electron dispersive spectroscopy were employed to characterize the morphology and structure of the CNFs. It has been found that the catalyst (Fe) thickness affected the morphology of the CNFs on the CF, resulting in different capacitive behaviors of the CNF/CF electrodes. Two different Fe thicknesses (5 and 10 nm) were studied. The capacitance behaviors of the CNF/CF electrodes were evaluated by cyclic voltammetry measurements. The highest specific capacitance, approximately 140 F g^−1^, has been obtained in the electrode grown with the 5-nm thickness of Fe. Samples with both Fe thicknesses showed good cycling performance over 2,000 cycles.

## Background

Electrochemical capacitors, also known as supercapacitors or ultracapacitors, are energy storage systems that differ from regular capacitors in that they have ultrahigh capacitance, long cycle life, and high power density [1-3]. Supercapacitors have many applications ranging from hybrid automobiles and large industrial equipment to storage devices for solar cells and portable consumer electronics [3,4]. Supercapacitors can be divided into two categories: electrical double-layer capacitors (EDLCs) and pseudocapacitors. In EDLCs, different forms of carbon are commonly used as active electrode materials, and the capacitance results from electrostatic charge accumulations at the electrode/electrolyte interfaces [5-7]. In contrast, in redox or pseudocapacitors, in which transition metal oxides such as RuO_2_·*x*H_2_O and MnO_2_ and electronically conducting polymers such as polyaniline and polypyrrole are used as active electrode materials [8-11], charge storage results from fast and reversible faradic reactions at the surface of the electroactive materials. Among the many candidates for supercapacitor electrode materials, mesoporous carbon spheres [12], carbon nanotubes (CNTs) and/or carbon nanofibers (CNFs) [13-16], CNT/polypyrrole composites [17], and MnO_2_/CNT composites [18] have attracted much attention due to their excellent electrical conductivity, large surface area, chemical inertness, and high operating temperature range. Several methods have been developed to synthesize CNTs and CNFs including arc discharge, laser ablation, and chemical vapor deposition (CVD) [19-21]. In the CVD process, transition metals such as nickel (Ni), cobalt (Co), iron (Fe), or their combination are used as the catalyst and are often deposited onto the substrates before the CNTs and CNFs are grown [22]. Then, carbon-containing precursor gases such as methane (CH_4_) [23], acetylene (C_2_H_2_) [24], ethylene (C_2_H_4_) [25], or ethane (C_2_H_6_) [26] with the carrier gases (argon and/or hydrogen) are introduced into the CVD system and decompose at the catalyst sites to form CNTs or CNFs at the corresponding gas decomposition temperature.

In the water-assisted chemical vapor deposition (WA-CVD), water vapor is introduced during the CVD process to enhance CNT/CNF growth [27]. Two main contributions of the water vapor are as follows: (1) it inhibits catalyst nanoparticles formed at CVD temperature from diffusing into the substrates by oxidizing metal nanoparticles such as Fe; (2) it removes amorphous carbon that is formed on the active catalyst surface, thereby increasing the catalyst lifetime [28].

Compared to commonly used silicon substrates, weaved carbon fabric (CF) has several advantages such as flexibility, scalability, light weight, and low cost. In addition, due to its weave structure, it has more surface area than other conventional substrates and is more advantageous for supercapacitor applications. In recent studies, active carbon [29], multi-walled carbon nanotubes (MWCNTs) [30-33], single-walled carbon nanotubes [34], CNT and polypyrrole composites [35], TiO_2_/MWCNTs [36], and graphene [37] have been successfully incorporated into the CF via various growth methods for supercapacitor applications.

In this work, CNFs are grown on CF substrates using the aforementioned WA-CVD method with Fe as the catalyst and C_2_H_4_ as the precursor gas. Furthermore, the effect of the CNF morphology on the capacitive performance is discussed. Scanning electron microscopy (SEM), transmission electron microscopy (TEM), and energy dispersive spectroscopy (EDS) are utilized to characterize the structure and morphology of the CNFs. The capacitive behaviors of the CNF/CF electrodes are investigated by cyclic voltammetry (CV) via a three-electrode system in a neutral aqueous Na_2_SO_4_ electrolyte solution.

## Methods

### CF material

Panex 30 carbon fabrics made from spun yarn (plain weaved; density, 1.75 g cm^−3^; thickness, 406 μm) were purchased from Zoltek (St. Louis, MO, USA). The fabrics were PAN-based materials that are >99% carbonized.

### Synthesis of the CNFs on CF

First, a thin film of Fe was deposited onto the CF substrate via DC sputtering at a base pressure of 10^−5^ Torr. The deposition rate of Fe was about 1.25 Å/s (RF power, 50W).The thickness of the Fe catalyst can significantly affect CNF morphology and distribution [38]. Two thicknesses (5 and 10 nm) of the Fe catalyst layer were deposited, and their influence on CNF morphology was compared.

**Figure 1:**
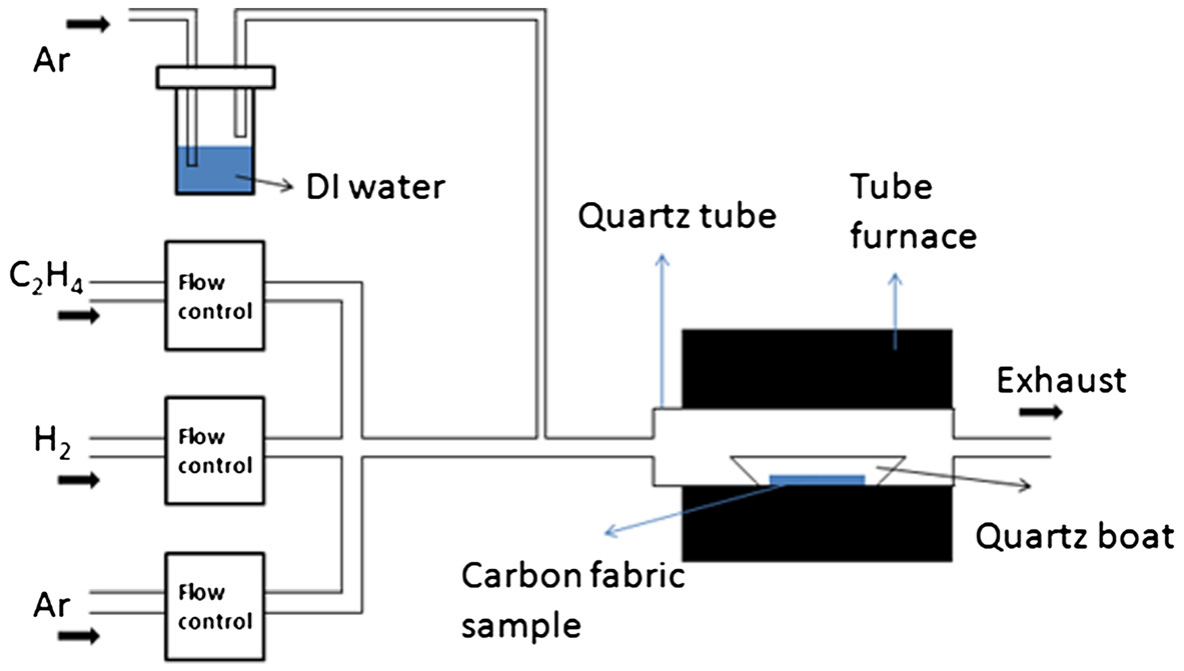
Schematic design of the WA-CVD process setup.

The CNFs were synthesized in a tube furnace via the WA-CVD method at 800°C as reported earlier [39]; however, a brief description is given as below. The experimental setup is shown in Figure 1. The CF samples with two different thicknesses of the Fe catalyst layer were first placed into a quartz boat that was placed in a quartz tube inside the furnace. Mass flow controllers allow Ar and H_2_ (500 and 10 sccm, respectively) to be introduced into the tube furnace to create an oxygen-free environment for CNF growth. In a previous study [40], it was found that the optimal gas flows (as shown in the Figure 1) for CNF growth are as follows: Ar, 200 sccm; H_2_, 10 sccm; and C_2_H_4_, 20 sccm. Additional Ar was introduced through a bubbler containing DI water (50 sccm). After flushing the tube furnace for 0.5 h, Ar flow was reduced to 200 sccm, and the furnace was heated to and maintained at 800°C for 10 min. Subsequently, 20 sccm of C_2_H_4_ was introduced. After 3 min, 50 sccm of Ar was introduced through the bubbler to deliver water molecules into the CNF growth system. The CNF growth time was 2 h; after which, both the C_2_H_4_ and water vapor flows were turned off. Ar flow was increased to 500 sccm to prevent oxygen from entering the CVD system. Then, the furnace was gradually cooled to ambient temperature. Lastly, the Ar and H_2_ were turned off before the samples were taken out.

### Characterization of the CNFs/CF

The Fe catalyst nanoparticle formation on the CFs as well as the morphology of the CNFs was investigated using a Zeiss Supra 55 field emission scanning electron microscope (Carl Zeiss AG, Oberkochen, Germany). EDS provides information for elemental analysis. The microstructure of the CNFs was studied by TEM using JEOL JEM 2100 F (JEOL Ltd., Akishima, Tokyo, Japan). The crystallinity of the CNFs was observed by electron diffraction (ED). A Sartorius CPA225D microbalance (Sartorius AG, Göttingen, Germany) with a resolution of 0.01 mg was used to measure the weight of the CNFs for the specific capacitance calculation. Before electrochemical measurement, the grown CNFs were treated with nanostrip (commercial mixture of concentrated H_2_SO_4_ and H_2_O_2_) to remove the remaining Fe catalyst particles to accurately measure the intrinsic capacitance of the CNFs on CF. Electrochemical measurements were carried out using Solartron SI 1287 electrochemical interface system (Solartron Analytical, Farnborough, UK) via a three-electrode configuration using the CNFs/CF as the working electrodes, a platinum plate as the counter electrode, and standard saturated calomel electrode as the reference electrode. A 0.5 M Na_2_SO_4_ aqueous neutral solution was used as the electrolyte. Cyclic voltammetry was performed over the potential range from −0.2 to 0.5 V at scan rates ranging from 5 to 100 mV s^−1^. Cycling tests were also conducted using the same configuration in order to investigate the specific capacitance behavior over 2,000 cycles.

## Results and discussion

### Morphology and structure of CNFs/CF

**Figure 2:**
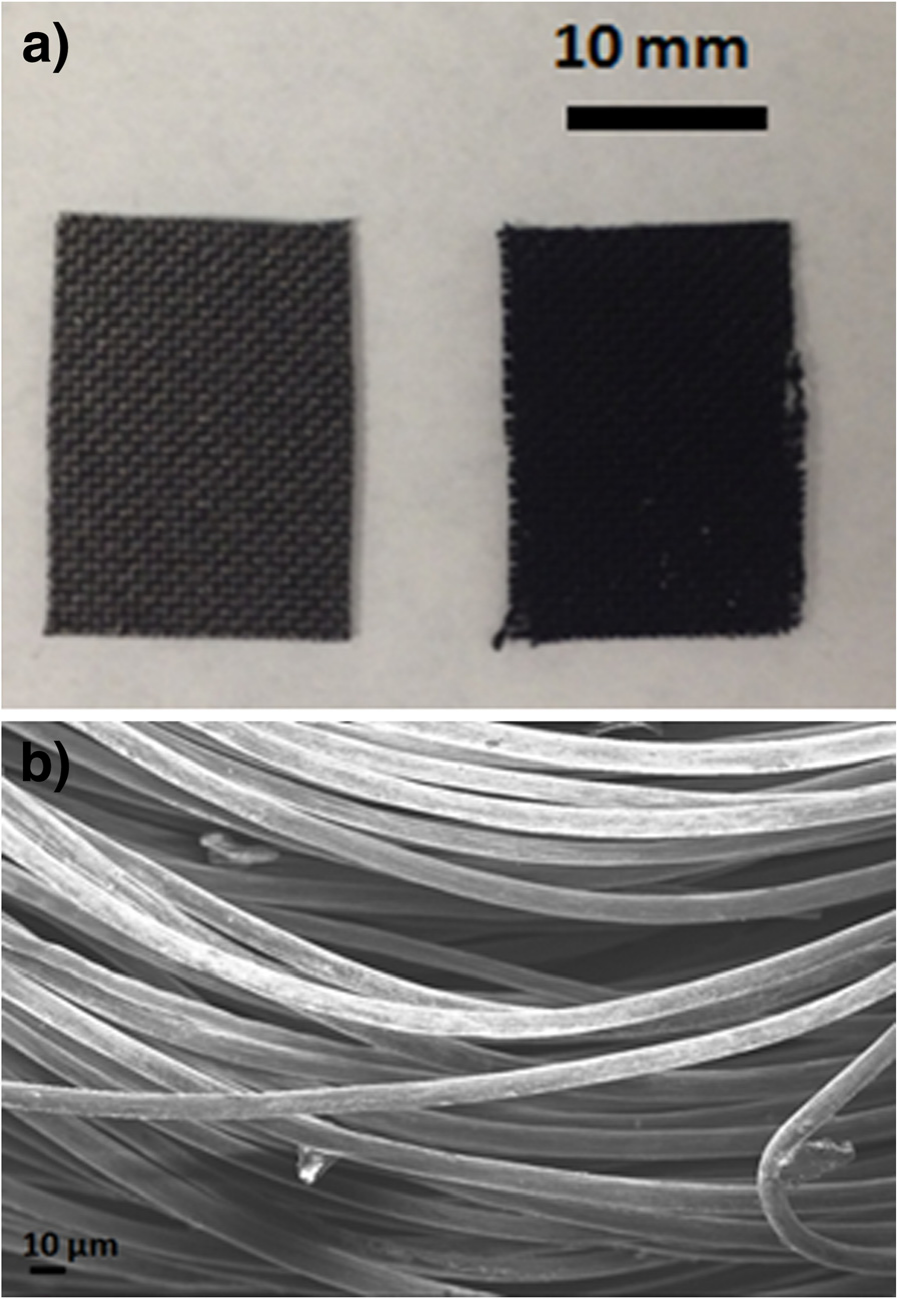
**CF, CNFs on CF, and CF fibrils.** (**a**) Photograph of pristine CF (left) and CNFs on CF (right). (**b**) SEM image of the CF fibrils before CNF growth.

After CNF growth, a black coating was observed on the CF as shown in Figure 2a which compares the pristine CF without CNFs with CNFs/CF. It can be seen that after CNF growth, the color of the CF became darker and the fabric structure was not apparent. Figure 2b shows the SEM image of the pristine CF, and it can be noted that the CF is composed of many individual fibrils with a diameter of 8 to 10 μm.

**Figure 3:**
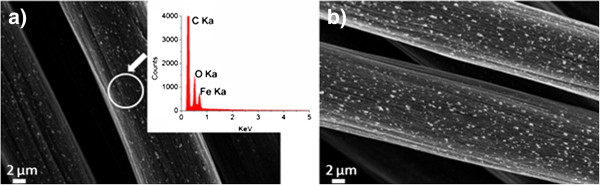
**SEM images of two thicknesses of Fe catalyst on CF after annealing.** (**a**) 5 nm of Fe catalyst on CF after annealing with the inset showing the EDS analysis of the circled area and (**b**) 10 nm of Fe catalyst on CF after annealing.

As discussed above, catalyst thickness can significantly affect the morphology and distribution of the CNFs. Thus, two catalyst thicknesses (5 and 10 nm) were deposited onto the CF to study the catalyst nanoparticle distribution. Two samples were heated in the tube furnace in an Ar and H_2_ environment at 800°C without the introduction of C_2_H_4_ to simulate the intermediate step of the formation of Fe nanoparticles from the Fe layer during the CVD process. Different morphologies and distributions of catalyst nanoparticles on the CF were observed by SEM as shown in Figure 3. Figure 3a shows an annealed 5-nm Fe catalyst layer deposited onto the CF with the inset showing an EDS analysis of the circled region; Figure 3b shows an annealed 10-nm Fe catalyst layer after the same process. The Fe and O peaks suggest that the nanoparticles are actually Fe_2_O_3_ which is due to exposure to air after the Fe sputtering, while the C peak is mainly from the CF. EDS analysis of the sample with 10 nm of Fe is similar to that with 5 nm of Fe; thus, it is not shown here. It can also be seen that samples with 10 nm of Fe have a denser distribution of Fe_2_O_3_ nanoparticles on the CF.

**Figure 4:**
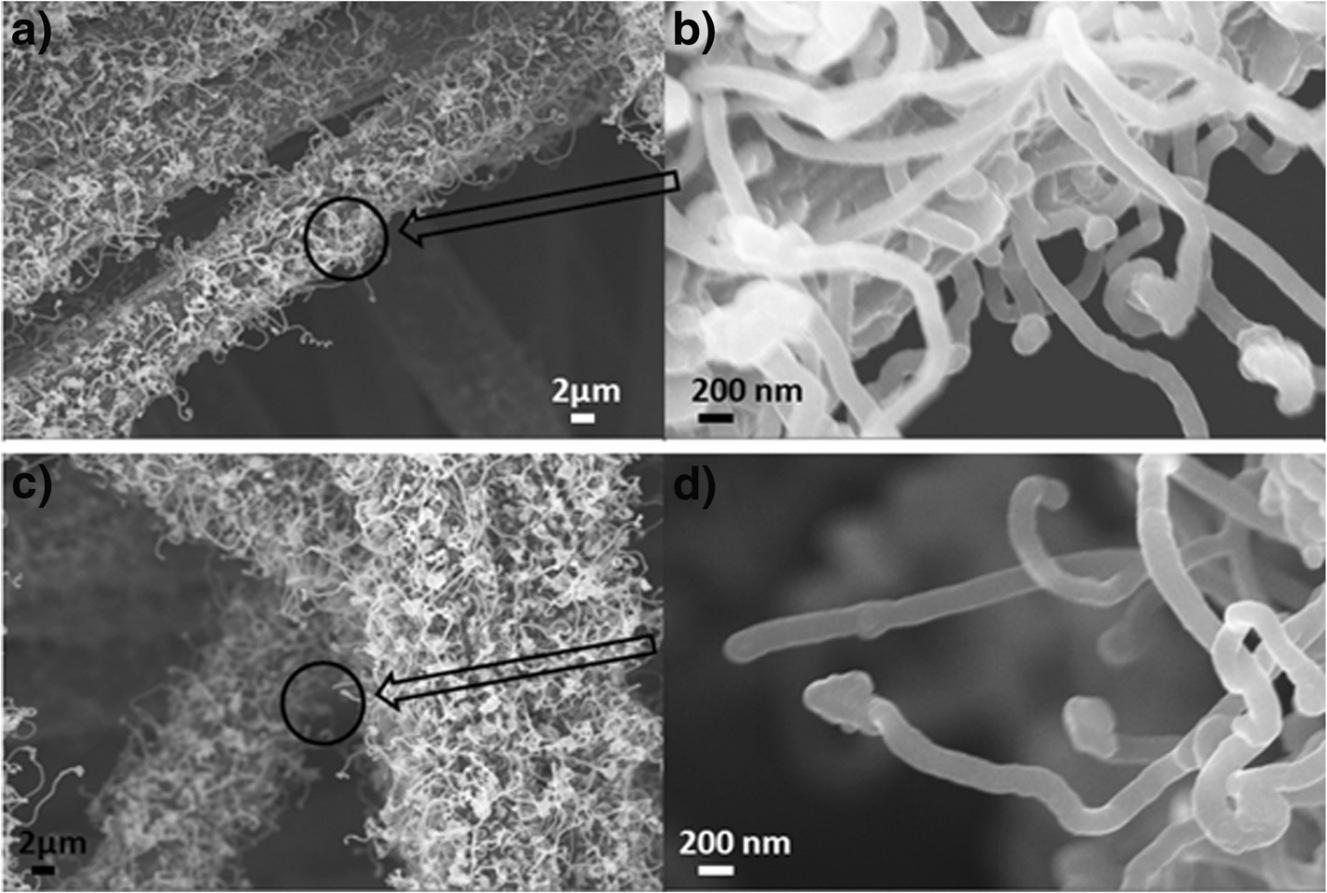
**SEM images of the CNFs on CF with different thicknesses of the Fe catalyst layer.** (**a**) CNFs grown with 5 nm of Fe and (**b**) higher magnification of the circled area in (**a**). (**c**) CNFs grown with 10 nm of Fe on CF and (**d**) higher magnification of the circled area in (**c**).

Figure 4 shows SEM images of the CNFs on CF with different thicknesses of the Fe catalyst layer. It is consistent with the nanoparticle distribution wherein the sample with 10 nm of Fe has denser CNF growth compared to that with 5 nm of Fe. Both images show entangled CNFs. It can also be seen from the higher magnification images (Figure 4b,d) that they are round shaped at the tips of the CNFs. EDS analysis (not shown here) revealed that they are the Fe catalyst particles. Although there has been a lot research progress on CNTs and CNFs since their discovery, the growth mechanism is not fully understood. The widely accepted growth mechanism of CNFs has the following steps: (1) decomposition of the precursor carbon-containing gases at the catalyst sites, (2) carbon incorporation into the catalysts, (3) saturation of the carbon and then precipitation out of the metal catalysts, and (4) formation of the carbon nanostructures [41,42]. There are commonly two growth modes of CNFs: the tip-growth model [43] and the base-growth model [44]. The tip-growth model results from a relatively weak catalyst-substrate interaction, and as they grow, the CNFs push the catalysts off the substrate, leaving the catalyst particles at the tips [45]. The base-growth model results from a relatively strong catalyst-substrate interaction with the catalyst particles remaining on the substrate [45]. In our work, given that the catalyst particles remain at the tips of the CNFs in both SEM and TEM images, the growth model of the CNFs is the tip-growth model.

**Figure 5:**
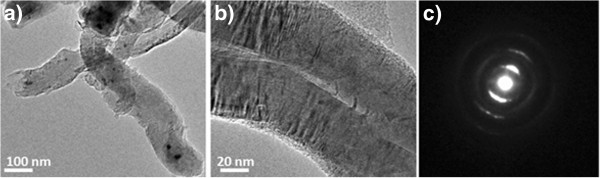
**TEM images and ED pattern of CNFs.** (**a**) TEM of the CNFs, (**b**) high-resolution TEM of the CNFs, and (**c**) electron diffraction pattern of the CNFs.

The detailed structure information of the CNFs is illustrated in the TEM images and the ED pattern as shown in Figure 5. The TEM images in Figure 5a,b demonstrate that the carbon nanostructures are CNFs as opposed to CNTs since the structures are solid rather than having a hollow inside that is typical for CNTs. These structures are likely to be caused by the catalyst-substrate interaction as discussed elsewhere [46]. The diameters of the CNFs ranged from 100 to 120 nm. From Figure 5b, it can be noticed that there are some dark lines across the CNFs perpendicular to their long axis; these are most likely the defects in the CNFs. The electron diffraction pattern in Figure 5c shows Debye rings, which indicates that the CNFs have polycrystallinity.

### CNF/CF properties as supercapacitor electrodes

The as-grown CNFs were treated with nanostrip for 2 h to remove the Fe catalyst particles so that the calculated specific capacitances are exclusively from CNFs/CF. It is also noticed that the CNFs/CF changed from hydrophobic to hydrophilic as a result of the nanostrip treatment. This is because the acid attacks the defects in the CNFs, forming carboxylic groups on the sidewalls as well as at the tip [47,48].

The specific capacitances were determined from the CV curves by the following equations [49-51]:

(1) $$C_{p}=\frac{q_{a}+\left|q_{c}\right|}{2m\Delta E}=\frac{\int_{E_{1}}^{E_{2}}i_{a}\left(E\right)dE+\left|\int_{E_{2}}^{E_{1}}i_{c}\left(E\right)dE\right|}{2m\Delta E}$$

where *C*_p_ is the specific capacitance, *m* is the mass of the CNFs, *ΔE* is the potential range, *q*_a_ and *q*_c_ are the anodic and cathodic charges during the positive and negative going scan, *i*_a_ and *i*_c_ are the anodic and cathodic currents, and *E*_1_ and *E*_2_ are the switching potentials of the CV.

Specific capacitance can be affected by many factors such as specific surface area, pore size, and conductivity [52,53]. However, these factors are interrelated, and a trade-off is usually needed when optimizing the specific capacitance. For instance, a small pore size may provide a large specific surface area, but it may also slow the diffusion of the electrolyte ions at interface; CNTs have less defects which leads to higher conductivity than CNFs, but the specific area of CNTs is much less than that of CNFs [52]. In this case, it is desirable for the carbon supercapacitor materials to have relatively high conductivity and also mesopores that are large enough for the electrolyte ions to diffuse and small enough to provide a large surface area [54].

**Figure 6:**
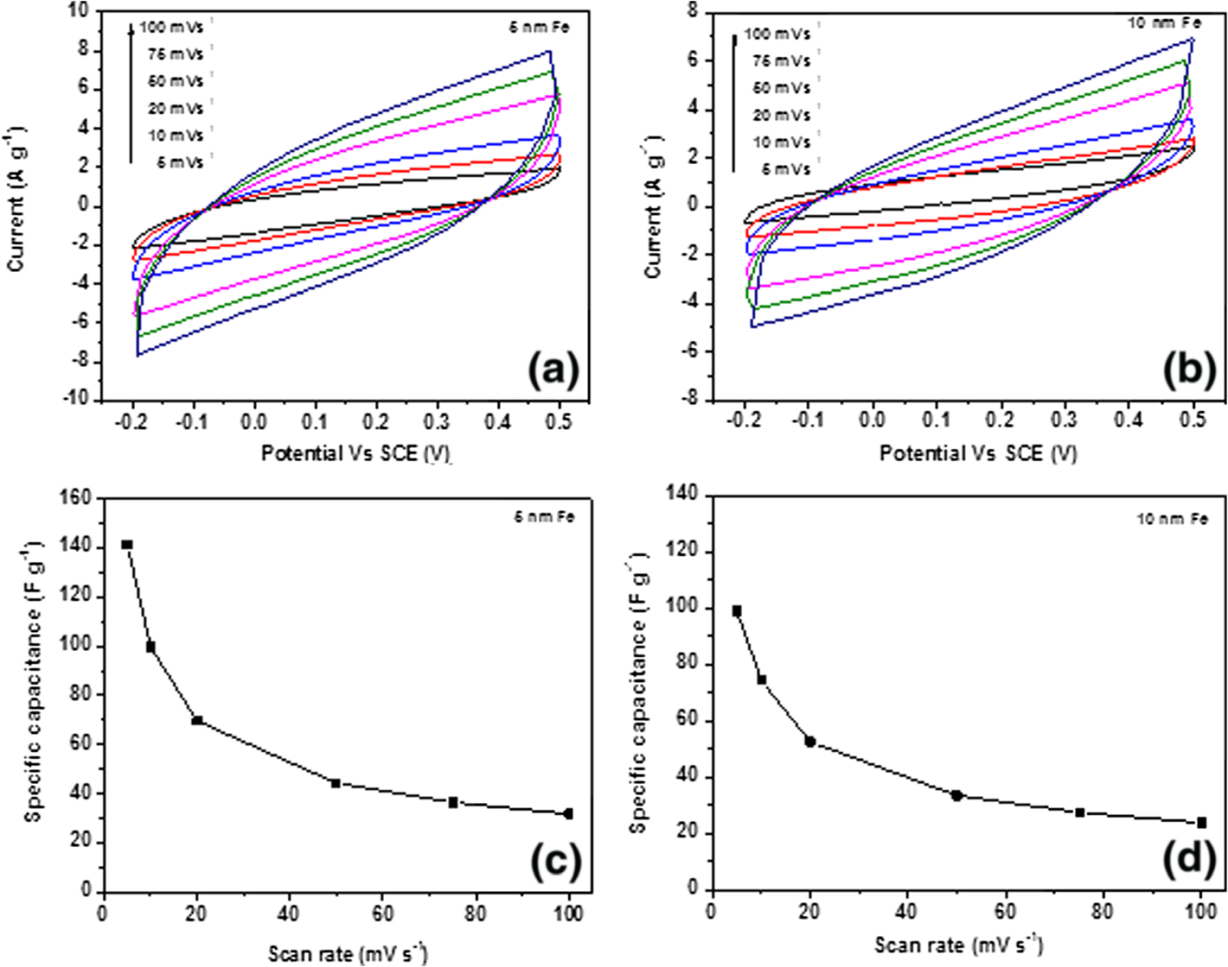
**CV curves and specific capacitances of CNF/CF electrodes.** (**a**) CV curves of a CNF/CF electrode (5 nm of Fe) at various scan rates. (**b**) CV curves of a CNF/CF electrode (10 nm of Fe) at various scan rates. (**c**) Specific capacitances of a CNF/CF electrode (5 nm of Fe) at various scan rates. (**d**) Specific capacitances of CNF/CF electrode (10 nm of Fe) at various scan rates.

Figure 6a,b shows the CV curves of the CNF/CF electrode grown with 5 and 10 nm of Fe catalyst via a three-electrode configuration at a potential window from −0.2 to 0.5 V in 0.5 M Na_2_SO_4_ at different scan rates. Both CNF/CF samples with different catalyst thicknesses (5 and 10 nm) exhibit good electrochemical performance. Both CV curves in Figure 6 are quasi-rectangular shape, which represent capacitive behaviors of CNF/CF electrodes. The areas of the close loop of the curves represent the charges stored at the CNFs/CF for one cycle. It is interesting to see that under the same scan rate, the charges stored at the CNFs grown with 5 nm of Fe are larger than those grown with 10 nm of Fe, which represent better capacitive behavior. Figure 6c,d shows the corresponding specific capacitances for different scan rates. For CNFs/CF with 5 nm of Fe, the specific capacitances are 142 and 32 F g^−1^ at the scan rates of 5 and 100 mV s^−1^, respectively. By comparison, for the CNF/CF electrode grown with 10 nm of Fe, the specific capacitances are 99 and 24 F g^−1^ at the scan rates of 5 and 100 mV s^−1^, respectively. For both samples, the specific capacitances at 100 mV s^−1^ only retained about 30% of the capacitances at 5 mV s^−1^. As stated above, this is likely to be related to the morphology of the entangled CNF structures which might hinder the diffusion ability of the electrolyte ions to travel from the aqueous solution to the electrode. It is also interesting to see that although the CNFs grown with 10 nm of Fe had a denser distribution of CNFs as suggested by Figure 4; the specific capacitance of the CNFs does not benefit from it. This suggests that the increasing density of the CNFs does not necessarily increase the specific area (area per unit gram) of the CNFs, and/or it can also decrease the pore size due to the higher degree of entanglement and thus lead to the attenuation of the electrolyte ion diffusion.

**Figure 7:**
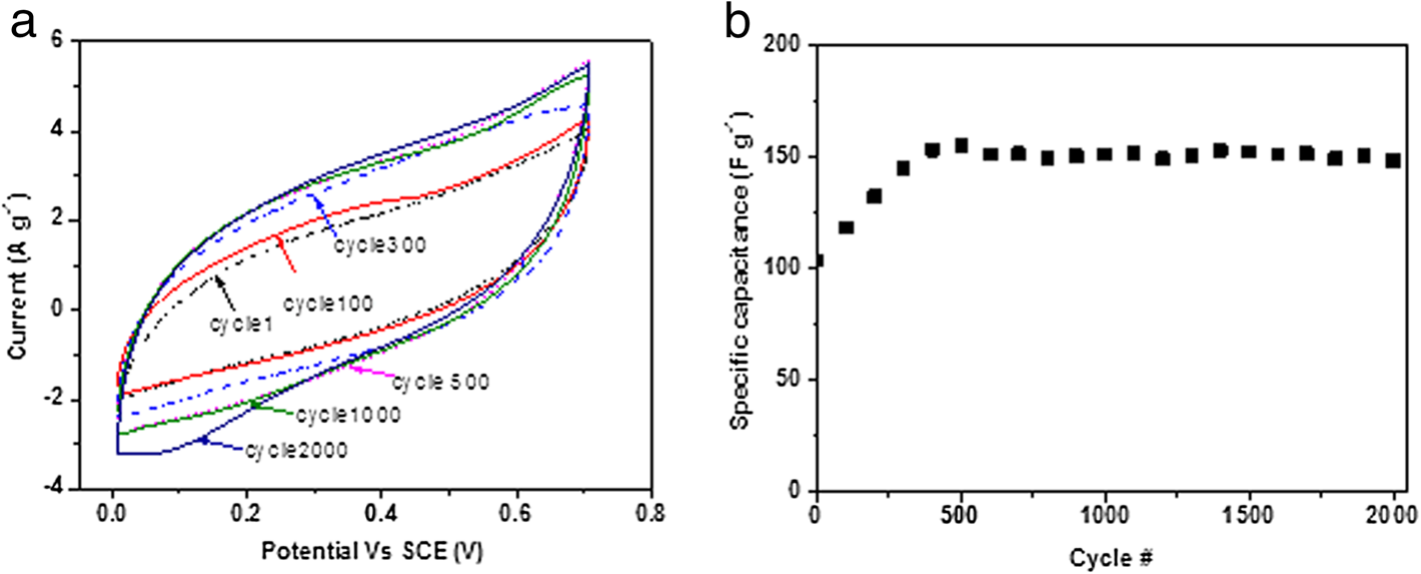
**CV results of a CNF during cycling test and specific capacitances.** (**a**) CV curves of a CNF/CF electrode (5 nm of Fe) during the cycling test. (**b**) Specific capacitances of a CNF/CF electrode as function of the number of cycles.

Figure 7a shows the CV results of the CNF grown with 5 nm of Fe during the cycling test, and Figure 7b shows the calculated specific capacitances for different cycles. It can be noted that in the first 500 cycles, the specific capacitance increased from 100 to 155 F g^−1^ and then started to stabilize at approximately 150 F g^−1^ for more than 2,000 cycles. The initial increase of the specific capacitance is also observed in other researchers' work [55,56] and is due to the activation process that may gradually let the trapped electrolyte ions diffuse out [57]. More importantly, the specific capacitance at the 2,000th cycle maintained approximately 95% of the peak capacitance value (155 F g^−1^ at the 500th cycle), which demonstrates a very good stability in the cycling performance of the CNF/CF electrodes.

## Conclusions

CNFs were directly grown on flexible CF substrates via the WA-CVD method using Fe as the catalysts and C_2_H_4_ as the precursor gas. Different thicknesses of the catalyst (5 and 10 nm) led to different morphologies and densities of the CNFs on the CF, thus resulting in different capacitive performances of the CNF/CF electrode as a supercapacitor. CNFs grown with 5 nm of Fe demonstrated better capacitive behaviors with a specific capacitance of approximately 140 F g^−1^ at the scan rate of 5 mV s^−1^, compared to 99 F g^−1^ for its counterpart. The electrode shows good cycling stability for more than 2,000 cycles. The CNF/CF electrodes are flexible, stretchable, and scalable, and hence, they could be a good candidate for flexible supercapacitor applications.

## Competing interests

The authors declare that they have no competing interests.

## Authors’ contributions

YG carried out the synthesis and the characterization of CNFs on carbon fabric and drafted the manuscript. GPP carried out electrochemical characterization of the CNFs/CF, made substantial contributions to the analysis and interpretation of data, and revised the draft of the manuscript critically. JT, CRW, and BG read and contributed to the improvement of the manuscript. All authors read and approved the final manuscript.
